# Delayed Care in Self-Presenting Stroke Patients: Real-World Data on Process Delays—Comparing Process Times Between Emergency Medical Services and Self-Presentation

**DOI:** 10.3390/jcm15072755

**Published:** 2026-04-06

**Authors:** Christian Claudi, Patrick Schramm, Martin Juenemann, Norma J. Diel, Tobias Fruehwald, Hendrik Loesche, Patrick Mueller-Nolte, Hagen B. Huttner, André Worm

**Affiliations:** 1 Department of Neurology, University Hospital Giessen, 35392 Giessen, Germany; 2 Department of Emergency Medicine, University Hospital Giessen, 35392 Giessen, Germany; 3 Department of Neurology, University Hospital Dresden, 01307 Dreseden, Germanyandre.worm@ukdd.de (A.W.); 4 Malteser Education Center Hesse, Rhineland-Palatinate and Saarland, 35578 Wetzlar, Germany; 5 Department of Emergency Medicine, Asklepios Stadtklinik Bad Wildungen, 34537 Bad Wildungen, Germany

**Keywords:** stroke, emergency medical services, self-presentation, process times, thrombolysis, public awareness, stroke education

## Abstract

**Background:** Stroke is a time-critical emergency in which rapid diagnosis and treatment are essential to improve outcomes. While Emergency Medical Services (EMS) facilitate structured prehospital stroke care, a proportion of patients bypass EMS and self-present to emergency departments (EDs), potentially causing treatment delays. This study compares process times and outcomes between EMS-transported and self-presenting stroke patients. **Methods:** We conducted a retrospective analysis of prospectively collected data from 1805 patients with suspected stroke admitted between May 2019 and June 2021 to two hospitals in Germany. Patients were classified as EMS-transported or self-presenting. Process times included door-to-needle time (DNT), symptom onset to first medical contact (FMC), and intravenous thrombolysis (IVT) rates. Data are presented as mean ± standard deviation or median [interquartile range] for continuous variables and as number (%) for categorical variables. **Results:** A total of 1940 patients with suspected stroke were included. For the main analysis comparing EMS transport and self-presentation, 1805 patients (1525 EMS, 280 self-presenting) were evaluated. EMS patients were significantly older than self-presenting patients (73.1 ± 15.1 vs. 65.3 ± 14.9 years, *p* < 0.001). The median time from symptom onset to first medical contact was 114 [95–132] minutes in EMS patients and 727 [420–1440] minutes in self-presenting patients (*p* < 0.001). IVT was administered in 119/1197 (9.9%) EMS patients and 18/254 (7.1%) self-presenting patients (*p* = 0.158) among those with documented information on thrombolysis. Door-to-needle time was slightly but significantly longer in self-presenting patients (median 35 [32–54] vs. 30 [28–34] minutes, *p* < 0.001), while both groups remained well within certification targets. **Conclusions:** Self-presenting stroke patients experienced markedly longer prehospital delays and lower intravenous thrombolysis rates compared to EMS-transported patients, although the difference in IVT rates was not statistically significant. In-hospital door-to-needle times were comparable between groups. These findings highlight the need for targeted public education to improve stroke symptom recognition and timely EMS activation.

## 1. Introduction

Stroke is a medical emergency that requires immediate diagnostics and therapy. It is a time-critical condition in which the effectiveness of reperfusion therapies decreases rapidly with increasing delay from symptom onset to treatment [1,2,3]. Early recognition and initiation of treatment are critical to achieving optimal patient outcomes.

Emergency Medical Services (EMS) play a crucial role in the diagnosis and treatment of stroke patients, ensuring timely and efficient care. By shortening the time from symptom onset to hospital assessment and enabling early initiation of stroke pathways, EMS activation is a key prerequisite for patients to reach reperfusion therapies in time [4]. Well-coordinated collaboration between EMS and hospitals, including pre-notification protocols, enables rapid diagnostics and treatment by ensuring that resources are allocated even before the patient’s arrival.

While the majority of stroke patients are initially evaluated and transported by EMS [5,6], a substantial proportion arrive at the hospital without EMS involvement. Studies indicate that up to 80% of stroke patients are assigned to hospitals by EMS [7]; although this figure derives from a Swedish study, similar proportions have been suggested for Germany [8].

In many emergency departments, patients not referred by EMS are triaged by nursing staff using standardized systems such as the Manchester Triage System (MTS) and the Emergency Severity Index (ESI) [9,10]. These systems, which typically allow a maximum waiting time of 10 min until the first physician contact for neurological deficits, may not always assign the most appropriate urgency to self-presenting stroke patients, potentially leading to additional delays in diagnostics and treatment [10].

When patients self-present, often due to a misinterpretation of symptoms or an underestimation of stroke severity, the initiation of diagnostic processes may be delayed, potentially worsening patient outcomes [11]. The aim of this substudy, which is part of a prospective evaluation of a new score for identifying stroke patients [8], is to investigate whether these theoretical considerations occur in clinical practice and to assess their relevance. Understanding how different modes of presentation influence these time-critical pathways is therefore essential for identifying targets for improvement in acute stroke care.

The objective of this study was to quantify the proportion of stroke patients who presented directly to emergency departments (EDs) without the assistance of Emergency Medical Services (EMS) and to compare their in-hospital process times with those of patients transported by EMS. By analyzing these differences, the study sought to determine whether self-presentation is associated with delays in the initiation of diagnostics and treatment, thereby providing evidence to inform targeted interventions aimed at reducing such delays.

## 2. Materials and Methods

This study is a retrospective subanalysis of prospectively collected data from a multicenter observational cohort (the FAST4D study) conducted between May 2019 and June 2021 [8]. The prospective parent study was approved by the institutional review board of the Justus-Liebig-University Giessen, Germany (Approval No. AZ 215/18, 18 January 2019) prior to patient enrollment, and conducted in accordance with the Declaration of Helsinki. Informed consent was waived because the study involved no interventions or changes to patient care, and data collection was performed independently of the study. The study was carried out in two counties in Germany (combined population approximately 565,000) and included patients recruited from two hospitals equipped with neurology departments, certified stroke units, and general medicine departments. Neither hospital offered mechanical thrombectomy; patients requiring this intervention were referred to nearby thrombectomy centers.

Patients presenting with suspected stroke, whether via Emergency Medical Services (EMS) or as self-referrals, were included in the analysis. This approach encompassed individuals ultimately discharged with a diagnosis of stroke—both ischemic and hemorrhagic—as well as cases in which stroke was initially suspected, thereby capturing stroke mimics relevant to the research question.

The analysis focused on key in-hospital process times. Two primary metrics were examined: door-to-needle time (DNT), defined as the interval from emergency department (ED) admission to initiation of intravenous thrombolysis (IVT), and the time from symptom onset to first medical contact (FMC). FMC was defined as the time of first assessment by a physician or paramedic after symptom onset. For EMS-transported patients, FMC corresponded to the documented time of first EMS contact; for self-presenting patients, FMC corresponded to the first physician contact in the emergency department. For patients assigned via EMS, the onset-to-FMC interval was extracted from EMS reports; for self-presenting patients, this information was obtained from ED records.

IVT use was recorded as a binary variable (yes/no); IVT rates were analyzed in patients with complete documentation of this variable.

Data collection was performed in accordance with the principles of the Declaration of Helsinki, and all patient information was pseudonymized. The study protocol adhered to the STROBE guidelines for observational research. Datasets with incongruent or missing critical information—such as absent process times, interhospital transfers, or cases already admitted as inpatients—were excluded from the analysis.

Data were analyzed descriptively. Continuous variables are reported as mean ± standard deviation when normally distributed and as median [IQR 25th–75th percentile] when non-normally distributed. Categorical variables are presented as counts and percentages. Group comparisons (EMS vs. self-presentation) were performed using the t-test or Mann–Whitney U test for continuous variables and chi-square or Fisher’s exact test for categorical variables, as appropriate. All analyses were conducted with SPSS (Version 25; IBM, New York, NY, USA); two-sided α = 0.05.

Generative artificial intelligence tools were used only for language editing (grammar and style) and were not involved in study design, data analysis, or interpretation of results.

## 3. Results

**Figure 1:**
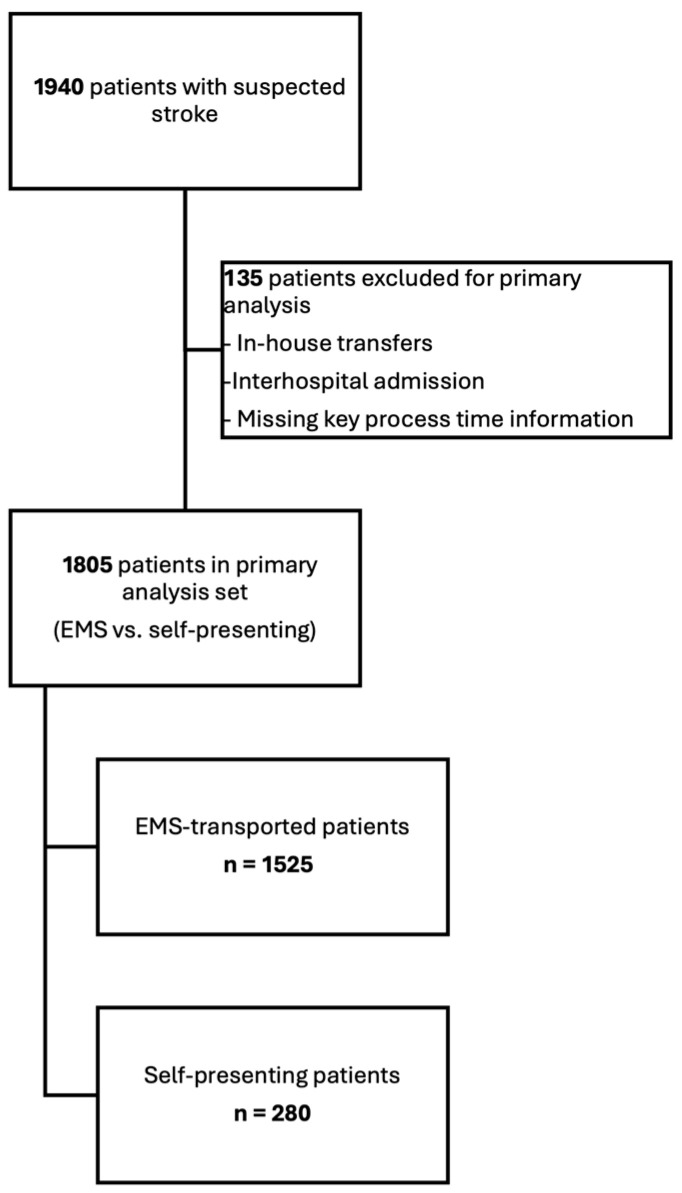
Patient flow. Flowchart illustrating the inclusion of 1940 patients with suspected stroke, the application of exclusion criteria (in-house transfers, interhospital admissions and missing key process time information) and the derivation of the primary analysis set of 1805 patients (1525 EMS-transported, 280 self-presenting). In total, 135 patients were excluded based on these criteria.

A total of 1940 patients were included in the analysis. For the primary comparison between EMS-transported and self-presenting patients, we analysed a subset of 1805 patients after excluding in-house transfers and interhospital admissions. Within this analysis set, 1525 patients (84.5%) arrived via EMS and 280 (15.5%) self-presented to the emergency department. The flow of patients through the study, including exclusions and derivation of the analysis set, is shown in Figure 1.

The overall mean age was 72.2 ± 15.1 years (95% CI 71.5–72.8), with 939 (48.4%) male and 1001 (51.6%) female patients. Baseline characteristics of EMS-transported and self-presenting patients are summarized in Table 1.

Patients were categorized by mode of admission as follows: 1525 (78.6%) were transported via Emergency Medical Services (EMS), 280 (14.4%) presented as self-referrals, and the remaining cases were in-house transfers or admissions from other hospitals. For analyses focusing solely on EMS versus self-presenting patients, a subset of 1805 patients were considered, excluding in-house transfers and interhospital admissions, reflecting real-world referral patterns in the participating regions.

Within this subset, 1322/1805 (73.2%) had a confirmed cerebrovascular event, while 483/1805 (26.8%) did not. Among those with a confirmed cerebrovascular event (*n* = 1322), 884 (66.9%) were classified as cerebral ischemia, 360 (27.2%) as transient ischemic attack (TIA), and 79 (6.0%) as intracranial hemorrhage (ICH). In the EMS group, the mean age was 73.1 ± 15.1 years, whereas in the self-presenting group it was 65.3 ± 14.9 years (*p* < 0.001).

Crosstabulation by gender showed 716 males and 809 females in the EMS group, and 153 males and 127 females in the self-presenting group (χ^2^ test, *p* = 0.018).

Process time metrics were evaluated next. In the EMS group, the median time from symptom onset to first medical contact was 114 min (IQR 95–132), the median time for in-hospital management was 49 min (IQR 48–50), and the median door-to-needle time was 30 min (IQR 28–34). In the self-presenting group, the median time from symptom onset to first medical contact was 727 min (IQR 420–1440) and the median door-to-needle time was 35 min (IQR 32–54). The difference in onset-to-first-medical-contact times between EMS and self-presenting patients was highly significant (*p* < 0.001), as was the difference in door-to-needle times (*p* < 0.001)

Information on the use of intravenous thrombolysis (IVT) was available for 1451 of the 1805 patients (80.4%). IVT rates were therefore calculated in this subset, comprising 1197 EMS-transported and 254 self-presenting patients. Among the 1451 patients with documented information on systemic thrombolysis, intravenous thrombolysis (IVT) was administered to 137 patients overall. In the EMS group, IVT was performed in 119/1197 (9.9%), while in the self-presenting group, IVT was performed in 18/254 (7.1%) (*p* = 0.158). Thus, the difference in IVT rates between EMS and self-presenting patients did not reach statistical significance.

Outcome measures using the modified Rankin Scale (mRS) are presented in Table 1 and Table 2.

At admission (see Table 2), self-presenting patients showed a more favorable distribution of mRS scores compared to EMS patients, with higher proportions in mRS 0–2 and fewer patients in mRS 4–5 (χ^2^ test, *p* < 0.001).

A similar pattern was observed at discharge (see Table 3), where self-presenting patients more frequently achieved mRS 0–2 and rarely had severe disability (mRS 4–5) compared with EMS patients (χ^2^ test, *p* < 0.001).

**Figure 2:**
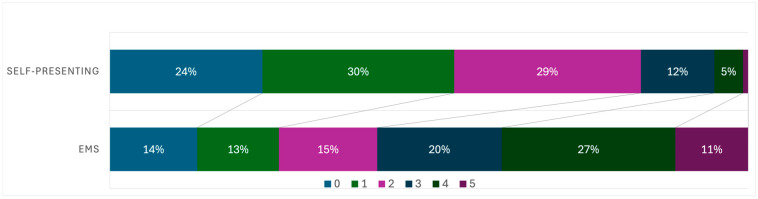
Distribution of modified Rankin Scale (mRS) scores at admission in patients transported by Emergency Medical Services (EMS) and in self-presenting patients. Bars show the proportion of patients in each mRS category (0–6) for EMS (*n* = 1525) and self-presenting patients (*n* = 280). The self-presenting group shows a higher proportion of patients with mRS 0–2 and fewer patients with mRS 4–5 compared with the EMS group. Group differences in the distribution of mRS scores are described in the Results (chi-square test).

**Figure 3:**
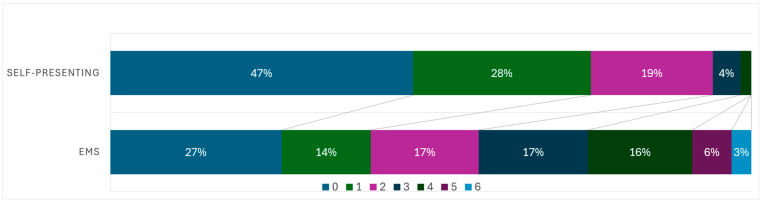
Distribution of modified Rankin Scale (mRS) scores at discharge in patients transported by Emergency Medical Services (EMS) and in self-presenting patients. Bars show the proportion of patients in each mRS category (0–6) for EMS (*n* = 1525) and self-presenting patients (*n* = 280). Self-presenting patients more frequently achieve mRS 0–2 and only rarely have severe disability (mRS 4–5), whereas EMS patients show a higher proportion of poor functional outcomes at discharge. Group differences in the distribution of mRS scores are described in the Results (chi-square test).

Figure 2 and Figure 3 visualize the mRS distributions at admission and discharge, respectively, and descriptively confirm the higher proportion of patients with mRS 0–2 and the lower proportion with mRS 4–5 in the self-presenting group.

## 4. Discussion

This study provides critical insights into the prolonged time intervals experienced by self-presenting stroke patients compared to those transported via Emergency Medical Services (EMS). Despite the significant advancements in EMS protocols and prehospital stroke care, substantial delays persist in patients who self-present, emphasizing the importance of targeted interventions to reduce these delays.

The mean age of patients in this study was 72.2 years, with a slight majority of female patients (51.6%). However, the proportion of male patients was higher among self-presenting patients than among EMS-transported patients (54.6% vs. 47.0%; χ^2^ test, *p* = 0.018). This difference suggests that male patients may be more likely to choose self-presentation instead of activating EMS; however, this association should be interpreted cautiously, as our observational design does not allow causal conclusions regarding sex-specific differences in health-seeking behaviour or risk perception. Overall, the demographic distribution aligns well with data reported in other studies on cerebrovascular events [12,13,14]. Such consistency reinforces the generalizability of the findings, as the study population reflects typical characteristics observed in stroke patients across different settings.

Among the patients with ischemic stroke included in this study, IVT was administered to EMS-transported patients at a rate of 9.9% and to self-presenting patients at a rate of 7.1%, without a statistically significant difference between groups. Overall, the IVT rate in this study was slightly below the national average of 16.3% but within the wide regional range of 2.9% to 32.0% reported in 2019 [14]. The door-to-needle time (DNT) was 30 min for EMS patients and 35 min for self-presenting patients, with both values well below the 60 min maximum specified by stroke unit certification criteria [15]. These findings suggest that while in-hospital processes are well-optimized, delays in prehospital care—particularly among self-presenting patients—remain a critical barrier to timely treatment. The slightly longer DNT in self-presenting patients may also reflect organizational factors, as triage and initial assessment in the emergency department are included in DNT for these patients, whereas EMS-transported patients often benefit from pre-notification and targeted routing that bypasses standard triage pathways. This underlines the need to ensure that in-hospital triage and recognition pathways reliably identify walk-in stroke patients as time-critical emergencies, so that they can access the same streamlined workflows as pre-notified EMS patients.

One of the most striking findings of this study is the substantial difference in the time from symptom onset to first medical contact (FMC) between EMS-transported patients and self-presenting patients. While the median interval for EMS patients was 114 min (approximately 1.9 h), self-presenting patients experienced a median delay of 727 min (approximately 12.1 h). This large difference in FMC times closely mirrors the high proportion of patients documented as being outside the therapeutic time window for IVT, particularly among self-presenting patients. This variability in prehospital time aligns with prior studies, such as the investigation by Helwig et al. [16], which highlighted significant delays in EMS activation following stroke onset. These findings underscore the critical need to address the recognition and timely response to stroke symptoms among the general public.

Although self-presenting patients showed more favorable mRS distributions at both admission and discharge, this should not be interpreted as evidence of a beneficial effect of self-presentation on functional outcome. This pattern is also evident in the graphical representation of mRS distributions at admission and discharge (Figure 1 and Figure 2), which depict a shift towards lower disability categories in the self-presenting group. Rather, these differences are best understood as reflecting baseline selection rather than any causal benefit of self-presentation, since self-presenting patients were younger, had milder deficits at presentation, and reperfusion rates were not higher in this group. The relatively favourable functional outcomes observed in self-presenting patients despite longer onset-to-first-medical-contact times are therefore likely explained by this more favourable baseline profile, including younger age, lower premorbid disability and a higher proportion of minor strokes, rather than by the timing of treatment alone.

Our results are consistent with earlier findings, including those from a multicentric study in 2002, which reported that only one-third of stroke patients reached a hospital within three hours of symptom onset [17]. Although technological advancements, extended treatment windows, and improved EMS protocols have enhanced stroke care over the years, the delayed presentation of self-referred patients highlights an enduring gap. Differences in regional healthcare systems, stroke awareness levels, and EMS accessibility may partially explain these disparities.

The prolonged delays in self-presenting patients suggest a lack of awareness regarding the critical nature of stroke symptoms. Public education campaigns, such as the “FAST” initiative, have demonstrated success in improving recognition of stroke symptoms and reducing response times [18]. The European Resuscitation Council’s “Kids Save Lives” initiative, as outlined in the position statement by Böttiger et al., may serve as a model for implementing school-based stroke education programs [19]. By incorporating stroke symptom recognition training into school curricula, children can act as catalysts for prompt EMS activation within their communities. Evidence from studies in the United States also supports the efficacy of such programs, showing that school-based education significantly improves public awareness and shortens the time to EMS activation [20,21].

Our results underscore the urgent need for targeted public health initiatives to enhance stroke symptom recognition and ensure prompt EMS activation. In addition to broad educational campaigns, tailored community-based interventions should focus on behaviorally high-risk groups, particularly younger and less severely affected patients who are more likely to self-present and delay EMS activation, while also addressing specific barriers faced by older patients where relevant. Moreover, policymakers and healthcare providers need to explore strategies to streamline the EMS referral process and improve accessibility, particularly in rural areas.

Based on the pronounced delay between symptom onset and first medical contact in self-presenting patients, we propose a novel hypothesis that regular training of schoolchildren on stroke symptom recognition may reduce delays in EMS activation. This hypothesis warrants further investigation through interventional studies, potentially providing a scalable solution to address the persistent prehospital delays observed in self-presenting stroke patients.

### Study Limitations

This study has several strengths, including a large sample size (1940 patients), the use of real-world data, and comprehensive process time metrics that allow for a detailed comparison between EMS-transported and self-presenting stroke patients. However, this study is not without limitations. The retrospective design inherently limits causal inferences, and the reliance on previously collected data may introduce bias. Additionally, the exclusion of datasets with incongruent or missing information could skew the results. There were relevant baseline differences between groups, particularly with respect to age, which may confound associations between mode of presentation and outcomes. Regional differences in stroke awareness and EMS practices further limit the generalizability of our findings. In addition, our study was conducted in two primary stroke centres without on-site mechanical thrombectomy, with patients requiring endovascular treatment being referred to external comprehensive stroke centres, which may limit the generalizability to regions with different thrombectomy network structures. Moreover, data on IVT use were not available for all patients, so IVT rates had to be calculated in the subset with complete documentation, which may have introduced information bias. The marked difference in group sizes (1525 EMS vs. 280 self-presenting patients) reflects real-world referral patterns but results in unequal statistical power between groups, which may limit the precision of estimates in the smaller self-presenting cohort. In addition, no multivariable adjustment for baseline differences such as age or premorbid mRS was performed, which limits causal interpretation of group differences and may confound associations between mode of presentation and outcomes. Furthermore, NIHSS scores were not systematically available for this subanalysis, and no retrospective reconstruction from medical records was performed, which precludes NIHSS-based comparisons between EMS-transported and self-presenting patients. Finally, discharge destination (e.g., home, inpatient rehabilitation or nursing home) was not systematically available for this subanalysis, which precludes a comparison of discharge disposition between EMS-transported and self-presenting patients. Therefore, future studies should adopt prospective, multicenter designs to validate these results and address these limitations.

## 5. Conclusions

Approximately 15.4% of stroke patients self-present to emergency departments, experiencing significantly longer times to first medical contact and numerically lower intravenous thrombolysis rates compared to those transported via EMS. In particular, younger and less severely affected patients appear more likely to delay EMS activation, which may contribute to prolonged prehospital intervals and reduced access to time-sensitive treatments, with relevant socioeconomic implications when functional impairments persist. This disparity underscores the critical need for targeted public education campaigns and innovative interventions, such as school-based training programs, to address the enduring delays in stroke care.

## Figures and Tables

**Table 1:** Baseline characteristics.

Characteristic	EMS	Self-Presenting	*p*-Value
Age, years	73 ± 15	65 ± 15	<0.001
Male, sex, *n* (%)	716/1525 (47.0)	153/280 (54.6)	0.018
Cerebrovascular event, *n* (% of all admissions)	1056/1525 (69.2)	266/280 (95.0)	<0.001
Type of cerebrovascular event (only patients with event)			0.281
- Ischemic stroke, *n* (%)	709 (67.1)	175 (65.8)	
- TIA, *n* (%)	279 (26.4)	80 (30.1)	
- Intracranial hemorrhage, *n* (%)	68 (6.4)	11 (4.1)	

Baseline characteristics of patients presenting with suspected stroke, stratified by mode of admission (EMS-transported vs. self-presenting). The table summarizes demographic variables and the distribution of cerebrovascular events, including ischemic stroke, transient ischemic attack, and intracranial hemorrhage, in both groups. Baseline characteristics are reported for the analysis set of 1805 patients (EMS-transported and self-presenting), excluding in-house transfers and interhospital admissions.

**Table 2:** Distribution of mRS Scores at Admission.

Group	0	1	2	3	4	5	6
EMS	13.7%	12.8%	15.4%	19.5%	27.2%	11.4%	0.0%
Self-presenting	23.9%	30.0%	29.2%	11.5%	4.5%	0.8%	0.0%

This table shows the percentage distribution of modified Rankin Scale (mRS) scores at admission for patients admitted via Emergency Medical Services (EMS) and those who self-presented. The mRS is a scale used to measure the degree of disability or dependence in daily activities of individuals who have experienced a stroke. The table includes all mRS categories from 0 (no symptoms) to 6 (death).

**Table 3:** Distribution of mRS Scores at Discharge.

Group	0	1	2	3	4	5	6
EMS	27.0%	14.0%	17.0%	17.2%	16.4%	6.2%	3.1%
Self-presenting	47.2%	27.7%	19.0%	4.3%	1.7%	0.0%	0.0%

This table shows the percentage distribution of modified Rankin Scale (mRS) scores at discharge for patients admitted via Emergency Medical Services (EMS) and those who self-presented. The mRS is a scale used to assess the level of disability or dependence following a stroke, with scores ranging from 0 (no symptoms) to 6 (death).

## Data Availability

The data presented in this study are available on request from the corresponding author. The data are not publicly available due to privacy restrictions.

## Abbreviations

The following abbreviations are used in this manuscript:

CI	Confidence interval
DNT	Door-to-needle time
ED	Emergency department
EMS	Emergency Medical Services
ESI	Emergency Severity Index
FMC	First medical contact
ICH	Intracranial hemorrhage
IQR	Interquartile range
IVT	Intravenous thrombolysis
MTS	Manchester Triage System
mRS	Modified Rankin Scale
STROBE	Strengthening the Reporting of Observational Studies in Epidemiology
TIA	Transient ischemic attack
